# Intraocular pressure-lowering effects of ripasudil in uveitic glaucoma, exfoliation glaucoma, and steroid-induced glaucoma patients: ROCK-S, a multicentre historical cohort study

**DOI:** 10.1038/s41598-020-66928-4

**Published:** 2020-06-25

**Authors:** Akiko Futakuchi, Takeshi Morimoto, Yoko Ikeda, Hidenobu Tanihara, Toshihiro Inoue, ROCK-S study group collaborators, Makoto Aihara, Shogo Arimura, Takeo Fukuchi, Tomomi Higashide, Megumi Honjo, Masaru Inatani, Kenji Inoue, Kyoko Ishida, Makoto Ishikawa, Keiichiro Iwao, Hiroshi Kakimoto, Kazuhide Kawase, Akitoshi Kimura, Shigeru Kinoshita, Yoshiaki Kiuchi, Taiki Kokubun, Shigeto Maekawa, Kazunori Miyata, Kazuhiko Mori, Shunsuke Nakakura, Natsuko Nakamura, Makoto Nakamura, Toru Nakazawa, Kenichi Namba, Takashi Nishida, Nobuyuki Ohguro, Shinichiro Ohtani, Hideaki Okumichi, Mari Sakamoto, Akira Sawada, Minako Shiokawa, Chie Sotozono, Aki Suetake, Kazuhisa Sugiyama, Yuko Takemoto, Kana Tokumo, Goji Tomita, Satsuki Tsuzaki, Sachiko Udagawa, Morio Ueno, Yu Yokoyama, Takeshi Yoshitomi, Yuki Yuasa

**Affiliations:** 1 Department of Ophthalmology, Faculty of Life Sciences, Kumamoto University, Kumamoto, 860-8556 Japan; 2 Department of Clinical Epidemiology, Hyogo College of Medicine, Hyogo, 663-8501 Japan; 3 Oike-Ikeda Eye Clinic, Kyoto, 604-8006 Japan; 4 Department of Ophthalmology, Kyoto Prefectural University of Medicine, Kyoto, 602-0841 Japan; 5 Kumamoto University Hospital, Kumamoto, 860-8556 Japan; 6 Department of Ophthalmology, The University of Tokyo, Tokyo, 113-8655 Japan; 7 Department of Ophthalmology, Faculty of Medical Sciences, University of Fukui, Fukui, 910-1193 Japan; 8 Division of Ophthalmology and Visual Science, Graduate School of Medical and Dental Sciences, Niigata University, Niigata, 951-8520 Japan; 9 Department of Ophthalmology, Kanazawa University Graduate School of Medical Science, Ishikawa, 920-8641 Japan; 10 Inouye Eye Hospital, Tokyo, 101-0062 Japan; 11 Department of Ophthalmology, Toho University Ohashi Medical Center, Tokyo, 153-8515 Japan; 12 Department of Ophthalmology, Akita University Graduate School of Medicine, Akita, 010-8543 Japan; 13 Kengun-Sakuragi Eye Clinic, Kumamoto, 861-2101 Japan; 14 Department of Ophthalmology, Gifu University Graduate School of Medicine, Gifu, 501-1194 Japan; 15 Department of Frontier Medical Science and Technology for Ophthalmology, Kyoto Prefectural University of Medicine, Kyoto, 602-8566 Japan; 16 Department of Ophthalmology and Visual Science, Graduate School of Biomedical Sciences, Hiroshima University, Hiroshima, 734-8551 Japan; 17 Department of Ophthalmology, Tohoku University Graduate School of Medicine, Miyagi, 980-8574 Japan; 18 Miyata Eye Hospital, Miyazaki, 885-0051 Japan; 19 Department of Ophthalmology, Saneikai Tsukazaki Hospital, Himeji, 671-1227 Japan; 20 Division of Ophthalmology, Department of Surgery, Kobe University Graduate School of Medicine, 650-0017 Hyogo, Japan; 21 Department of Ophthalmology, Faculty of Medicine and Graduate School of Medicine, Hokkaido University, Hokkaido, 060-8638 Japan; 22 Department of Ophthalmology, Japan Community Health Care Organization, Osaka Hospital, Osaka, 553-0003 Japan; 23 Department of Orthoptics, Fukuoka International University of Health and Welfare, Fukuoka, 814-0001 Japan

**Keywords:** Glaucoma, Clinical trials

## Abstract

To evaluate the efficacy and safety of ripasudil for treatment of secondary glaucoma, a historical cohort study was conducted at 18 centres in Japan. Adults (age ≥20 years) who needed additional IOP reduction and received topical 0.4% ripasudil between 2014 and 2018 due to three secondary glaucoma subtypes, including uveitic glaucoma (UG), exfoliation glaucoma (EG) or steroid-induced glaucoma (SG) were assessed for mean IOP change from baseline prior to additional treatment with ripasudil. We further evaluated the IOP change in each glaucoma subtype, baseline characteristics of each cohort, course of uveitis-induced inflammation in UG eyes, and proportion of patients in each cohort with adverse events. In 332 eyes from 332 patients eligible for this study, the mean overall IOP reductions from baseline at 1, 3, and 6 months were −5.86 ± 9.04 mmHg (−19.4 ± 25.1%), −6.18 ± 9.03 mmHg (−20.0 ± 27.1%), and −7.00 ± 8.60 mmHg (−23.4 ± 25.6%), respectively. These changes were all statistically significant. Of 332 eyes, 109 eyes had UG, 181 had EG, and 42 eyes had SG. The IOP-lowering effects of ripasudil in UG and SG were significantly greater than those of EG at every time point. This finding could have been related to higher baseline IOP levels in UG and SG. UG patients exhibited significant decreases in mean cell score of the anterior segment after ripasudil treatment. No severe adverse events were reported. These findings suggest that treatment with ripasudil is a safe and effective therapeutic modality for IOP reduction in secondary glaucoma.

## Introduction

The Rho protein contributes to various physiological events in many organs and tissues. Rho and its effector molecule Rho-associated kinase (ROCK) are involved in diverse cellular functions, including stress fibre formation, focal adhesion, cell contraction, motility and polarity^1–3^. Rho/ROCK signalling molecules are present in the aqueous outflow pathway^4,5^, and regulate aqueous humour outflow, contributing to the pathologies of some subtypes of glaucoma^6,7^. Rho/ROCK inhibition lowers intraocular pressure (IOP) primarily by increasing aqueous humour outflow directly through the conventional pathway, which is comprised of the trabecular meshwork (TM) and Schlemm’s canal (SC)^8–11^. ROCK inhibitors have been shown to induce alterations in cell shape, contraction, motility, attachment and extracellular matrix production in TM and SC cells^10^. Moreover, several recent studies have demonstrated anti-inflammatory effects of ROCK inhibitors such as ripasudil^12,13^, including inhibition of immune cell infiltration and inflammatory cytokine production.

Because IOP reduction is the primary therapeutic strategy for inhibiting the onset and progression of glaucomatous optic neuropathy, the IOP-lowering mechanism of the ROCK inhibitor ripasudil suggests its utility as a novel glaucoma therapeutic agent. In 2014, ripasudil was approved as an intraocular pressure (IOP)-lowering medication^14^. The IOP-lowering mechanism of ROCK inhibitor is different from other commercially available anti-glaucoma medications, which suppress aqueous humor production or promote uveoscleral outflow^15^. Moreover, prolonged use of other existing antiglaucoma medications induced recruitment of inflammatory cells in the conjunctiva and Tenon’s capsule, suggesting their potential proinflammatory properties^16^, which may exacerbate inflammation in uveitic glaucoma (UG) or subconjunctival wound healing process on possible glaucoma filtration surgery glaucoma patients may undergo in the future. Thus, it is possible that ROCK inhibition could reduce IOP even in non- or weak responders to other anti-glaucoma medications. Indeed, a number of randomized clinical trials demonstrated that ripasudil effectively lowers IOP in patients with primary open-angle glaucoma and ocular hypertension^17–19^. However, clinical evidence of the IOP-lowering effects of ripasudil in patients with other glaucoma subtypes are far from satisfactory, so the efficacy of ripasudil in these subtypes should be evaluated.

Glaucoma is categorized into primary, secondary and developmental subtypes. Among these subtypes, secondary glaucoma arises from various ocular or systemic disorders or conditions that obstruct aqueous humour outflow and thus elevate IOP. A population-based epidemiological survey, the Japanese Tajimi Study, reported that the estimated prevalence of secondary glaucoma in Japanese population older than 40 years was 0.5%, which accounted for 10% of total incidence of glaucoma of 5%^20^. Secondary glaucoma is often refractory to conventional glaucoma treatments, usually due to the underlying disorders/conditions. Thus far, in small-scale retrospective clinical studies, ripasudil has been shown to reduce IOP in patients with secondary glaucoma, including UG and exfoliation glaucoma (EG)^21,22^. Although these studies suggested that ripasudil was safe and effective in these secondary glaucoma subtypes, the limited sample sizes and retrospective study designs limited their interpretation. Recently, we reported a large-scale post-marketing surveillance study of ripasudil, named the Ripasudil Observational study to Confirm the safety and efficacy of Rho Kinase inhibitor for long-term use in Japanese patients with glaucoma (ROCK-J study)^23^. This survey also suggested significant IOP-lowering effects of ripasudil in secondary glaucoma patients.

In the present study, we conducted a retrospective multicentre historical cohort study for patients diagnosed with UG, EG or steroid-induced glaucoma (SG), in 18 Japanese ophthalmology departments to elucidate the clinical characteristics of the IOP-lowering responsiveness to ripasudil in secondary glaucoma patients.

## Results

### Study population

A total of 361 patients with glaucoma secondary to uveitis, exfoliation, or steroid use were registered in this retrospective study. Twenty-nine patients were determined ineligible for this study primarily due to lack of sufficient IOP data, and 332 secondary glaucoma patients were eventually enrolled in the study (ROCK-S study: Ripasudil Observational study to Confirm the efficacy and safety of Rho Kinase inhibitor in Japanese patients with Secondary glaucoma). In the included patients, the mean (±SD) age at enrolment was 69 ± 14 years, and the majority of patients were male (180 of 332 patients, 54%). Demographic data are presented in Table 1. The study included a total of 332 eyes from 332 patients, including 109 (33%) eyes with UG, 181 (55%) eyes with EG and 42 (13%) eyes with SG. The age distribution, visual acuity, and pre-treatment IOP were significantly different between groups. The EG group had the oldest patients and the lowest IOP. Pre-treatment visual acuity was lowest in the UG group. However, there were no differences between groups in sex, visual field, lens status, or number of glaucoma medications.

**Table 1:** Baseline Demographics.

		All (n = 332)	UG (n = 109)	EG (n = 181)	SG (n = 42)	P-value between subtypes
Mean age, yrs (SD)		69 ± 14	63 ± 14	76 ± 8	55 ± 16	<0.0001
Gender, n (%)						
Male		180 (54%)	56 (51%)	99 (55%)	25 (60%)	0.65
Female		152 (46%)	53 (49%)	82 (45%)	17 (40%)	
Visual acuity, logMAR (SD)		0.19 ± 0.41	0.29 ± 0.50	0.15 ± 0.36	0.06 ± 0.22	0.02
MD, dB (SD)		−11.0 ± 9.6	−12.4 ± 9.7	−10.6 ± 9.6	−10.0 ± 9.2	0.50
PSD, dB (SD)		7.0 ± 4.3	7.5 ± 4.5	7.0 ± 4.3	5.8 ± 3.8	0.45
Lens status (phakia), n (%)		203 (61%)	63 (58%)	108 (60%)	32 (76%)	0.10
Baseline IOP, mmHg (SD)		23.8 ± 9.0	27.5 ± 10.0	21.0 ± 7.2	26.8 ± 9.3	<0.0001
Antiglaucoma eye drops at baseline (SD)		3.0 ± 1.2	2.9 ± 1.1	3.1 ± 1.3	2.7 ± 1.2	0.17

UG: uveitic glaucoma, EG: exfoliation glaucoma, SG: steroid-induced glaucoma, SD: standard deviation, IOP: intraocular pressure, MD: mean deviation, PSD: pattern standard deviation.

Clinical characteristics of UG patients are shown in Table 2. Of 109 UG eyes included in the study, 52 cases were granulomatous uveitis (48%), and 57 cases were non-granulomatous uveitis (52%). Causes of uveitis included sarcoidosis (n = 22, 20%), Vogt-Koyanagi-Harada (VKH) disease (n = 8, 7%), Behcet’s disease (n = 4, 4%), herpes simplex virus infection (n = 5, 5%), and others (n = 20, 18%). The cause of uveitis was unknown in the remaining 50 eyes (46%). The average duration (± SD) from uveitis diagnosis to ripasudil prescription was 63.6 ± 81.5 months, and the average peripheral anterior synechiae (PAS) index (± SD) was 18.1 ± 29.2%. In 89 (82%) of 109 UG eyes and 38 (90%) of 42 SG eyes, steroid therapies had been prescribed prior to ripasudil treatment. The detailed route of steroid administration in UG and SG patients are shown in Supplementary Table 1.

**Table 2:** Characteristics of UG patients.

		Number of eyes	Ratio, %
Type of uveitis	Granulomatous uveitis	52	48
	Non-granulomatous uveitis	57	52
Cause of uveitis	Sarcoidosis	22	20
	VKH disease	8	7
	Behcet’s disease	4	4
	herpes simplex virus infection	5	5
	others	20	18
	unknown	50	46
Primary site of inflammation	anterior	65	60
	intermediate	8	7
	pan-uveitis	36	33
Anterior chamber cell score	0	60	55
	0.5+	3	3
	1+	17	16
	2+	4	4
	3+	1	1
	unknown	24	22
Anterior chamber flare score	0	67	61
	0.5+	4	4
	1+	9	8
	2+	3	3
	unknown	26	24
Uveitis duration, months (SD)		63.6 ± 81.5	
PAS index, % (SD)		18.1 ± 29.2	

UG: uveitic glaucoma, VKH disease: Vogt-Koyanagi-Harada disease, SD: standard deviation, PAS: peripheral anterior synechiae.

### Efficacy

#### IOP time course in combined secondary glaucoma patients

**Figure 1:**
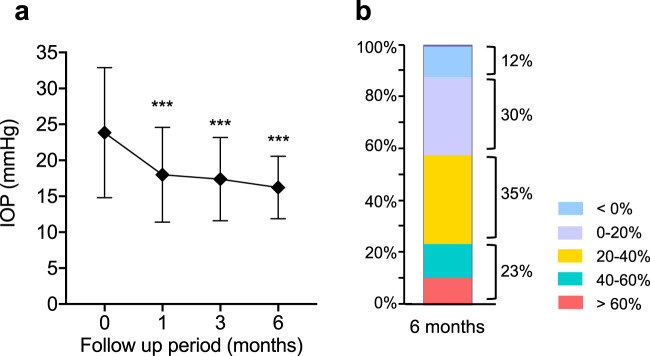
IOP-lowering effects of ripasudil in all secondary glaucoma patients. (**a**) Time-dependent changes in IOP over 6 months of follow-up after ripasudil treatment. Data are shown as IOP (mean ± SD) of maximum number of patients with no dropouts or missing data (n = 332 at 0 month, n = 273 at 1 month, n = 231 at 3 months, and n = 207 at 6 months, respectively). Statistical analysis was performed using IOP changes from the baseline. ***p < 0.0001. **(b)** Stacked bar chart of % IOP reduction from the baseline at 6 months after ripasudil treatment.

In all 332 eyes included in analysis, mean IOP reductions from baseline at 1, 3, and 6 months were significantly lower than baseline: −5.86 ± 9.04 mmHg (−19.4 ± 25.1%), −6.18 ± 9.03 mmHg (−20.0 ± 27.1%), and −7.00 ± 8.60 mmHg (−23.4 ± 25.6%), respectively, and these mean IOP changes were statistically significant (p < 0.0001, Fig. 1a). At the end of the study (6 months), 120 (58%) of all cases with no deficit in data (n = 207) achieved a greater than 20% IOP reduction from the baseline (Fig. 1b). Of those, 48 (23%) of the 207 eyes achieved a greater than 40% IOP reduction from baseline. Multiple regression analysis of data from 207 eyes with no deficit in data demonstrated that the IOP reduction at 6 months was significantly associated with baseline IOP (p < 0.0001), but not with age or lens status (Table 3).

**Table 3:** Relationship between IOP change at 6 months after ripasudil treatment and background factors determined by multiple regression analysis in all patients.

Background factor	T-value	P-value
Age (years)	1.29	0.20
Baseline IOP prior to ripasudil treatment (mmHg)	22.25	<0.0001
Lens status (phakia or not)	0.54	0.59

IOP: intraocular pressure.

#### IOP time course by glaucoma subtype

**Figure 2:**
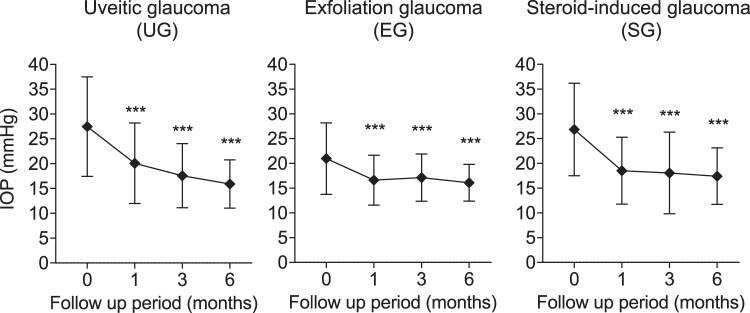
Time-dependent changes in IOP over 6 months of follow-up in each secondary glaucoma subtype after ripasudil treatment. Data are shown as IOP (mean ± SD) of maximum number of patients with no dropouts or missing data (UG, n = 109 at 0 month, n = 91 at 1 month, n = 69 at 3 months, and n = 58 at 6 months, respectively; EG, n = 181 at 0 month, n = 151 at 1 month, n = 134 at 3 months, and n = 123 at 6 months, respectively; SG, n = 42 at 0 month, n = 31 at 1 month, n = 28 at 3 months, and n = 26 at 6 months, respectively). Statistical analysis was performed using IOP changes from the baseline. ***p < 0.0001.

**Figure 3:**
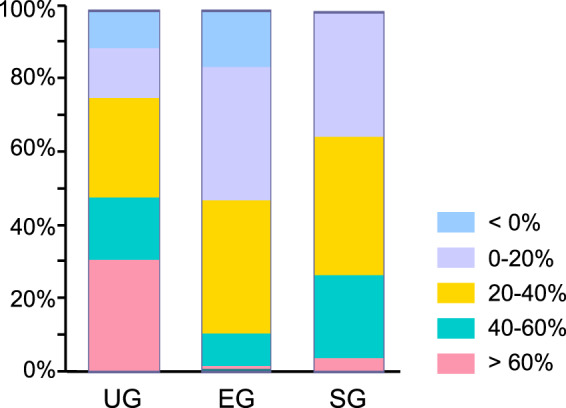
Stacked bar chart of %IOP reduction from the baseline 6 months after ripasudil treatment in each secondary glaucoma subtype.

Figure 2 represents the IOP time course of each secondary glaucoma subtype. Mean IOP reductions from baseline in the UG, EG and SG groups were −7.39 ± 11.02 mmHg (p < 0.0001), −4.25 ± 6.55 mmHg (p < 0.0001), and −9.26 ± 11.40 mmHg (p < 0.0001), respectively, mean IOP change rates: −21.2 ± 30.7%, −16.5 ± 20.6%, and −28.4 ± 25.3%, respectively, at 1 month, −10.04 ± 11.87 mmHg (p < 0.0001), −3.68 ± 6.46 mmHg (p < 0.0001) and −8.61 ± 7.66 mmHg (p < 0.0001), respectively, mean IOP change rates: −28.2 ± 33.9%, −13.7 ± 22.2%, and −29.7 ± 21.0%, respectively, at 3 months, and −12.59 ± 10.83 mmHg (p < 0.0001), −4.22 ± 6.14 mmHg (p < 0.0001), and −7.69 ± 6.86 mmHg (p < 0.0001), respectively, mean IOP change rates: −37.0 ± 28.9%, −16.1 ± 22.6%, and −28.0 ± 16.5%, respectively, at 6 months. Statistically significant differences in IOP reduction were present at every timepoint between three glaucoma subtypes (p = 0.0025 at 1 month, p < 0.0001 at 3 and 6 months); IOP reduction in the EG group was less than that of the other two subtypes. At the end of the study (6 months), 76% of UG eyes, 48% of EG eyes, and 65% of SG eyes achieved a greater than 20% IOP reduction from baseline (Fig. 3). Of those, 48% of UG eyes and 27% of SG eyes achieved a greater than 40% IOP reduction from baseline, while in EG eyes only 11% achieved this reduction. Multiple regression analysis of data from 30 UG eyes with no data deficits identified that the IOP reduction at 6 months was significantly associated with baseline IOP, but not with age, lens status, uveitis characteristics (granulomatous or non-granulomatous), PAS index, or cell score grading prior to ripasudil treatment (Table 4). Multiple regression analysis of data from 123 EG eyes with no deficits in data identified that IOP reduction at 6 months was significantly associated with baseline IOP, but not with age or lens status (Table 5). Multiple regression analysis of data from 25 SG eyes with no deficit in data identified that IOP reduction at 6 months was significantly associated with age, baseline IOP, and topical administration of steroids, but not with lens status (Table 6). The relationship between IOP change at 1 month after ripasudil treatment and background factors determined by multiple regression analysis in all patients, UG patients, EG patients, and SG patients are shown in Supplementary Tables 2–5.

**Table 4:** Relationship between IOP changes at 6 months after ripasudil treatment and background factors determined by multiple regression analysis in UG patients.

Background factor	T-value	P-value
Age (years)	0.04	0.97
Baseline IOP prior to ripasudil treatment (mmHg)	11.2	<0.0001
Lens status (phakia or not)	−0.59	0.56
Uveitis characteristics (granulomatous or not)	−0.05	0.96
PAS index (%)	−0.89	0.38
Cell score grading	0.55	0.59

IOP: intraocular pressure, UG: uveitic glaucoma, PAS: peripheral anterior synechiae.

**Table 5:** Relationships between IOP changes at 6 months after ripasudil treatment and background factors determined by multiple regression analysis in EG patients.

Background factor	T-value	P-value
Age (years)	−0.33	0.74
Baseline IOP prior to ripasudil treatment (mmHg)	15.69	<0.0001
Lens status (phakia or not)	0.14	0.89

IOP: intraocular pressure, EG: exfoliation glaucoma.

**Table 6:** Relationships between IOP changes at 6 months after ripasudil treatment and background factors determined by multiple regression analysis in SG patients.

Background factor	T-value	P-value
Age (years)	3.03	0.007
Baseline IOP prior to ripasudil treatment (mmHg)	4.41	0.0003
Lens status (phakia or not)	0.93	0.37
Topical administration of steroids	3.25	0.004

IOP: intraocular pressure, SG: steroid-induced glaucoma.

#### Course of uveitis activity

Table 7 shows changes in mean cell and flare scores in UG eyes over time. The average cell scores at 1, 3, and 6 months were significantly lower than the baseline: −0.16 ± 0.51 (p = 0.0163), −0.19 ± 0.44 (p = 0.0006), and −0.18 ± 0.55 (p = 0.0196), respectively. On the other hand, however, average flare scores from baseline, which was already low (average grade of 0.20), at 1, 3, and 6 months were not significantly different: −0.021 ± 0.32 (p = 0.4931), −0.05 ± 0.42 (p = 0.3308), and −0.02 ± 0.55 (p = 0.7482), respectively.

**Table 7:** Course of uveitis activity in UG patients.

Follow up periods (months)	Cell Scores		Flare Scores	
	Mean Changes in cell score from the baseline	P-value	Mean Changes in flare score from the baseline	P-value
1	−0.16 ± 0.51	0.0163	−0.021 ± 0.32	0.49
3	−0.19 ± 0.44	0.0006	−0.05 ± 0.42	0.33
6	−0.18 ± 0.55	0.0196	−0.02 ± 0.55	0.75

UG: uveitic glaucoma.

#### Subgroup analysis of low- and high-baseline IOP in EG eyes

**Figure 4:**
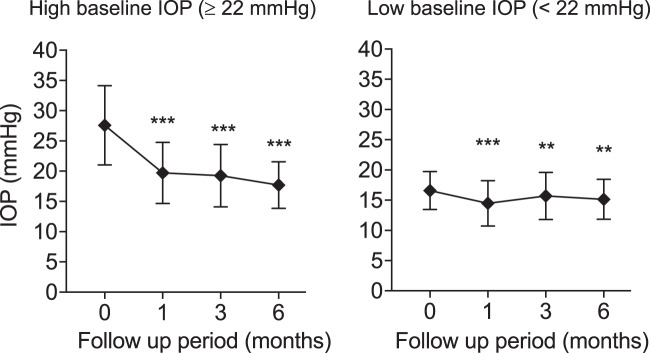
Subgroup analysis of EG eyes by baseline IOP. High baseline IOP; ≥22 mmHg, Low baseline IOP; <22 mmHg. Data are shown as IOP (mean ± SD) of maximum number of patients with no dropouts or missing data (High baseline IOP, n = 72 at 0 month, n = 62 at 1 month, n = 54 at 3 months, and n = 46 at 6 months, respectively; Low baseline IOP, n = 109 at 0 month, n = 89 at 1 month, n = 80 at 3 months, and n = 77 at 6 months, respectively). Statistical analysis was performed using IOP changes from the baseline. **p < 0.01 and ***p < 0.0001.

Multiple regression analysis suggested that the IOP-lowering effect of ripasudil was dependent on baseline IOP, and that the baseline IOPs of the three glaucoma subtypes were not equivalent, with the EG group being lowest. We thus performed subgroup analysis of EG eyes according to baseline IOP (high; ≥22 mmHg, n = 72, and low; <22 mmHg, n = 109). Mean IOP reductions from baseline in the high and low baseline IOP were −7.85 ± 8.35 mmHg (p < 0.0001) and −1.74 ± 3.04 mmHg (p < 0.0001), respectively, mean IOP change rates: −25.6 ± 20.9% and −10.2 ± 17.9%, respectively, at 1 month, −7.64 ± 7.78 mmHg (p < 0.0001) and −1.01 ± 3.41 mmHg (p = 0.0097), respectively, mean IOP change rates: −26.2 ± 19.8% and −5.2 ± 19.7%, respectively, at 3 months, and −8.87 ± 6.89 mmHg (p < 0.0001) and −1.44 ± 3.40 mmHg (p = 0.0004), respectively, mean IOP change rates: −31.3 ± 17.0% and −7.0 ± 20.5%, respectively, at 6 months (Fig. 4). Statistically significant differences in IOP reduction were present at every timepoint between high and low baseline IOP (p < 0.0001 at 1, 3 and 6 months). In the high-baseline IOP subgroup of EG eyes, IOP reduction rates were comparable to those of UG and SG eyes.

#### Adverse event incidence, severity, and subject discontinuation

The median follow-up period was 4.3 months for this 6-month retrospective study, with a 62.4% follow-up rate at 6 months. In 136 (37.7%) of 361 patients, at least one adverse event was reported (Table 8). A total of 152 adverse events were reported, and the most common adverse events were blurred vision (n = 27), followed by conjunctival hyperemia (n = 23), blepharitis (n = 10), eye pruritus (n = 10) and instillation site pain (n = 10). Severe adverse events were not reported for the duration of the study. Table 9 shows the detailed patient dropout data. Overall dropout rate was 25.0% (83 cases). In each glaucoma subtype, the dropout rates were 36.7% (40 cases) in UG patients, 16.6% (30 cases) in EG patients, and 31.0% (13 cases) in SG patients. The dropout rate due to inadequate IOP reduction in UG patients was 27.5% (30/109 cases), which was higher than those of EG patients (9.9%, 18/181 cases) and SG patients (19.0%, 8/42 cases).

**Table 8:** Adverse events in ROCK-S study.

Number of patients analyzed	361
Number of patients with adverse events	136
Number of adverse events	152
**Ocular events**	
Blurred vision	27
Conjunctival hyperemia	23
Blepharitis	10
Eye pruritus	10
Instillation site pain	10
IOP increased	9
Instillation site discomfort	6
Decreased visual acuity	4
Eyelid oedema	3
Conjunctival hemorrhage	2
Lacrimation increased	2
Instillation site irritation	2
Foreign body sensation	2
Double vision	2
Dry eye	2
Conjunctival follicles	2
Eyelid irritation	2
Eyelid pruritus	2
Photophobia	2
Abnormal sensation in eye	1
Keratitis filamentosa	1
Eye pain in the contralateral eye	1
Eye discharge	1
Blurry near focus	1
Poor visibility	1
**Nonocular events**	
Drowsiness	4
Feeling cramped	4
Dizziness	3
Chest discomfort	2
Fatigue	2
Numbness	2
Headache	1
Malaise	1
Constipation	1
Dysphonia	1
Others	3

IOP: intraocular pressure.

**Table 9:** Patient dropouts.

Reason for dropout	UG	EG	SG
Inadequate IOP reduction	30	18	8
Adverse events	3	11	4
Discontinuation of ripasudil for reasons other than adverse events	2	1	0
Cataract surgery	1	0	1
Low IOP	4	0	0
Total number of patient dropouts	40	30	13

IOP: intraocular pressure, UG: uveitic glaucoma, EG: exfoliation glaucoma, SG: steroid-induced glaucoma.

## Discussion

The present retrospective multicentre study provides large-scale data on the efficacy and safety of ripasudil in patients with secondary glaucoma, including UG, EG and SG. The study included 332 eyes with secondary glaucoma, consisting of 109 UG, 181 EG and 42 SG eyes. To the best of our knowledge, this is the largest patient cohort to be evaluated for the efficacy of ROCK inhibitors in secondary glaucoma. Our findings strongly support the IOP-lowering effects of ripasudil in patients with secondary glaucoma; in all 332 eyes included in the efficacy analysis, the mean IOP reductions from baseline at 1, 3, and 6 months were −5.86 ± 9.04 mmHg (−19.4 ± 25.1%), −6.18 ± 9.03 mmHg (−20.0 ± 27.1%), and −7.00 ± 8.60 mmHg (−23.4 ± 25.6%), respectively, which were all statistically significant. In addition, the study design allowed us to analyse the background factors related to ripasudil IOP reduction.

For EG, some previous reports, including ours, identified significant IOP-lowering effects of ripasudil^22,23^. Matsumura *et al*. reported that in 27 eyes from 16 patients with EG, ripasudil significantly lowered IOP. By contrast, some prior reports suggested no or minimal IOP-lowering effects for ripasudil in EG^24^. However, these negative results were derived from retrospective studies with small-scale case series. In the present study, our data from 181 EG eyes identified significant IOP reduction of 3.68–4.25 mmHg at every time point after ripasudil treatment. Our sub-analysis demonstrated a significant difference in the IOP-lowering effect of ripasudil dependent on baseline IOP. In EG cases with high baseline IOP (≥22 mmHg), the IOP-lowering effect of ripasudil (7.64–8.87 mmHg from baseline) was larger than the IOP-lowering effect of ripasudil (1.01–1.74 mmHg from baseline) in those with low baseline IOP (<22 mmHg).

For UG and SG, IOP reductions from baseline were significantly larger than those for EG. This finding could be related to higher baseline IOP levels. In 109 UG cases, the baseline IOP was 27.5 mmHg, and in 42 SG cases, the baseline IOP was 26.8 mmHg, whereas it was 21.0 mmHg in 181 EG cases, a significant difference. Additionally, in EG cases with high baseline IOP, the IOP-lowering effect of ripasudil was comparable with UG cases and SG cases as mentioned above. Our results indicated that IOP reduction after ripasudil treatment was associated with higher baseline IOP levels, which is consistent with prior reports^23^.

Moreover, in UG patients, the average anterior segment cell score was significantly decreased from baseline after ripasudil treatment, suggesting a possible anti-inflammatory effect of ripasudil in uveitis. Uchida *et al*. demonstrated the therapeutic effect of ripasudil in an endotoxin-induced rat uveitis model^12^, in which ripasudil inhibited LPS-induced nuclear translocation of Nuclear factor-kappa beta (NF-κB), adhesion molecules, and proinflammatory mediators, including monocyte chemotactic protein-1 (MCP-1), a key chemokine in chronically inflamed eyes^25^. Kusuhara *et al*. reported IOP-lowering effects of ripasudil in 21 UG eyes^21^, in which IOP reductions from baseline were -3, -4, and -4 mmHg at 1, 3, and 6 months, respectively, after ripasudil initiation. IOP reduction rates achieved in this prior study were smaller than those of the present study. This difference could be attributed to the higher baseline IOP in our study, or to the sample size. In addition, the state of primary uveitis should be taken into account when evaluating IOP in UG, as ripasudil treatment is likely to be initiated during the process of primary disease progression. Therefore, it is difficult to strictly distinguish the IOP-lowering effects of ripasudil in secondary glaucoma from the effect of recovery from primary uveitis.

In this study, the overall dropout rate was 25.0% (83 cases), including 36.7% (40 cases) in UG patients, 16.6% (30 cases) in EG patients, and 31.0% (13 cases) in SG patients. Notably, the dropout rate in UG patients due to inadequate IOP reduction was 27.5% (30/109 cases), which was higher than that of EG patients (9.9%, 18/181 cases) or SG patients (19.0%, 8/42 cases). Therefore, a possible limitation is the missing data of UG patients whose eyes were excluded due to insufficient drug efficacy, which could have had a considerable impact on the observed IOP-lowering effects of ripasudil in UG patients. In addition to the 30 dropout cases of UG due to inadequate IOP reduction, 6 cases were ineffective to ripasudil after 6 months of follow-up in UG patients (Fig. 3). We performed a comparative analysis of the characteristics of effective and ineffective UG patients, but there were no significant differences in age distribution, gender, lens status, baseline IOP levels, type of uveitis, causes of uveitis, primary site of inflammation, anterior chamber cell/flare score, uveitis duration, steroid administration, or PAS index. The underlying uveitis conditions could be confounders, but we could not perform additional analysis of more detailed uveitis conditions in this study due to its retrospective study design and limited sample size. Further clinical studies are required in the future to precisely elucidate the underlying pathology of secondary glaucoma in relation to the ripasudil effectiveness.

## Conclusions

The present historical large-scale study demonstrated favourable efficacy of ripasudil in three types of secondary glaucoma, UG, EG and SG. In UG and SG, the IOP-lowering effects of ripasudil were significantly larger than those of EG. This finding was likely related to the higher baseline IOP levels of UG and SG. In a subgroup analysis of EG eyes according to baseline IOP, IOP reduction rates of higher baseline IOP EG cases (≥22 mmHg) were comparable to those of UG and SG eyes. UG patients exhibited significant decreases in mean anterior segment cell scores after ripasudil treatment, suggesting that ripasudil has anti-inflammatory effects in primary uveitis. No severe adverse events were observed in this study.

## Methods

### Study design and patients

The study was a historical cohort study that enrolled secondary glaucoma patients prescribed topical ripasudil at 18 centres in Japan between 2014 and 2018 (Supplementary Table 6). The study was approved by the institutional review boards of Kumamoto University and participating centres, and adhered to the tenets of the Declaration of Helsinki and Japanese ethical guidelines for medical and health research involving human subjects. Informed consent from patients was substituted by the opt-out method. Inclusion criteria were: (1) patients 20 years or older and (2) patients that needed additional IOP reduction and received topical 0.4% ripasudil (Glanatec ophthalmic solution 0.4%, Kowa Company, Ltd., Japan) due to UG, EG or SG. Exclusion criteria were: (1) patients that chose to opt out of research, (2) patients contraindicated for usage of ripasudil, (3) patients with abnormalities of the anterior segment that affected Goldmann-applanation tonometry, (4) patients with any previous history of ocular surgery or laser treatment other than cataract surgery more than 6 months prior to the study, (5) patients with obscured view of the optic nerve, (6) patients with a history of ocular trauma, and (7) patients with optic nerve or intracranial disease that affected the visual field. In patients in whom both eyes fulfilled the inclusion criteria, the eye with higher baseline IOP was selected for analysis.

The primary outcome measure was mean IOP change from baseline. We also assessed the course of uveitis-induced inflammation activity in UG, and adverse events due to ripasudil in all groups.

### Data collection and statistical analysis

Patient characteristics were obtained from medical records. IOP was measured via Goldmann applanation tonometry, and was recorded prior to ripasudil treatment, and 1 month ± 1 week, 3 months ± 2 weeks, and 6 months ± 4 weeks after initiating ripasudil treatment. Dropout for any reason, including additional medication, surgery, or adverse events, was considered as study discontinuation. Data collection and management were independently conducted by the Institute for Clinical Effectiveness, Kyoto, Japan, using an electronic data capture (EDC) system.

Categorical variables are presented as numbers and percentages, and continuous variables are presented as either the mean and standard deviation or the median and IQR based on their distributions. Categorical variables were compared using the chi-squared test when appropriate; otherwise, the Fisher’s exact test was used. Change in continuous variables were assessed by paired t-test and compared using a t-test between two groups or an ANOVA between glaucoma subtypes. Multivariable linear regression models were constructed to assess associations between IOP changes at 1 month and 6 months after ripasudil treatment and background factors in all patients and in each glaucoma subtype. The course of uveitis activity in UG patients was assessed by Wilcoxon signed rank test. Bonferroni correction was utilized to compare the changes in sequential time course. All statistical analyses were centrally performed at the Institute for Clinical Effectiveness by a study statistician (T. Morimoto). All statistical analyses were conducted using JMP version 10.0.2 (SAS Institute Inc., Cary, NC, USA). All reported P-values were 2-tailed, and P-values <0.05 were considered statistically significant.

### Compliance with ethics guidelines

This study was conducted as a retrospective, multi-centre, historical cohort study. The study protocol was reviewed and approved by the ethics committees of the all participating medical institutions.

### Authorship

All named authors meet the International Committee of Medical Journal Editors (ICMJE) criteria for authorship, take responsibility for the integrity of the work as a whole, and have given their approval for the publication of the manuscript.

## Supplementary information

Supplementary information.

## Data Availability

The datasets generated during and/or analysed during the current study are available from the corresponding author on reasonable request.
