# Supplementary material for: Intraocular pressure-lowering effects of ripasudil in uveitic glaucoma, exfoliation glaucoma, and steroid-induced glaucoma patients: ROCK-S, a multicentre historical cohort study
Source: Sci Rep. 2020 Jun 25;10:10308. doi: 10.1038/s41598-020-66928-4 (PMC7316751; doi:10.1038/s41598-020-66928-4)
Supplement: Supplementary file 1 — Supplementary information. [file 41598_2020_66928_MOESM1_ESM.docx]

Supplementary Table 1. The route of steroid administration in UG and SG patients enrolled in this study

|  | UG (n=109) | SG (n=42) |
| --- | --- | --- |
| Topical | 75 (69%) | 16 (38%) |
| Systemic | 5 (5%) | 16 (38%) |
| Both | 9 (8%) | 6 (14%) |
| Total | 89 (82%) | 38 (90%) |

UG: uveitic glaucoma, SG: steroid-induced glaucoma

Supplementary Table 2. Relationship between IOP change at 1 month after ripasudil treatment and background factors determined by multiple regression analysis in all patients (Data from 273 eyes with no deficits in data).

| Background factor | T-value | P-value |
| --- | --- | --- |
| Age (years) | 0.28 | 0.78 |
| Baseline IOP prior to ripasudil treatment (mmHg) | 17.19 | <0.0001 |
| Lens status (phakia or not) | -0.56 | 0.58 |

IOP: intraocular pressure

Supplementary Table 3. Relationship between IOP change at 1 month after ripasudil treatment and background factors determined by multiple regression analysis in UG patients (Data from 45 eyes with no deficits in data).

| Background factor | T-value | P-value |
| --- | --- | --- |
| Age (years) | -0.39 | 0.70 |
| Baseline IOP prior to ripasudil treatment (mmHg) | 7.15 | <0.0001 |
| Lens status (phakia or not) | -1.16 | 0.25 |
| Uveitis characteristics (granulomatous or not) | -0.15 | 0.88 |
| PAS index (%) | -0.42 | 0.68 |
| cell score grading | 0.13 | 0.90 |

IOP: intraocular pressure, UG: uveitic glaucoma, PAS: peripheral anterior synechiae

Supplementary Table 4. Relationship between IOP change at 1 month after ripasudil treatment and background factors determined by multiple regression analysis in EG patients (Data from 123 eyes with no deficits in data).

| Background factor | T-value | P-value |
| --- | --- | --- |
| Age (years) | 2.87 | 0.005 |
| Baseline IOP prior to ripasudil treatment (mmHg) | 13.81 | <0.0001 |
| Lens status (phakia or not) | 0.94 | 0.35 |

IOP: intraocular pressure, EG: exfoliation glaucoma

Supplementary Table 5. Relationship between IOP change at 1 month after ripasudil treatment and background factors determined by multiple regression analysis in SG patients (Data from 29 eyes with no deficits in data).

| Background factor | T-value | P-value |
| --- | --- | --- |
| Age (years) | -1.39 | 0.18 |
| Baseline IOP prior to ripasudil treatment (mmHg) | 8.55 | <0.0001 |
| Lens status (phakia or not) | -2.39 | 0.03 |
| Topical administration of steroids | -1.11 | 0.28 |

IOP: intraocular pressure, SG: steroid-induced glaucoma

Supplementary Table 6. Number of enrolled patients in each facility.

|  | UG | EG | SG | Total |
| --- | --- | --- | --- | --- |
| Kumamoto University | 19 | 29 | 8 | 56 |
| Oike-Ikeda Eye Clinic | 12 | 43 | 3 | 58 |
| Kyoto Prefectural University of Medicine | 12 | 24 | 8 | 44 |
| Toho University Ohashi Medical Center | 9 | 19 | 5 | 33 |
| University of Fukui | 10 | 16 | 4 | 30 |
| Kobe University Graduate School of Medicine | 9 | 11 | 5 | 25 |
| Inouye Eye Hospital | 0 | 18 | 0 | 18 |
| Kanazawa University Graduate School of Medical Science | 8 | 4 | 2 | 14 |
| Niigata University | 8 | 0 | 2 | 10 |
| The University of Tokyo | 3 | 2 | 4 | 9 |
| Tohoku University Graduate School of Medicine | 4 | 4 | 0 | 8 |
| Hokkaido University | 8 | 0 | 0 | 8 |
| Japan Community Health Care Organization, Osaka Hospital | 5 | 0 | 0 | 5 |
| Saneikai Tsukazaki Hospital | 0 | 4 | 0 | 4 |
| Akita University Graduate School of Medicine | 1 | 2 | 0 | 3 |
| Miyata Eye Hospital | 0 | 3 | 0 | 3 |
| Gifu University Graduate School of Medicine | 0 | 2 | 0 | 2 |
| Hiroshima University | 1 | 0 | 1 | 2 |
| Total | 109 | 181 | 42 | 332 |

UG: uveitic glaucoma, EG: exfoliation glaucoma, SG: steroid-induced glaucoma,
